# Supplementary material for: A Systematic Review on Contamination of Marine Species by Chromium and Zinc: Effects on Animal Health and Risk to Consumer Health
Source: J Xenobiot. 2025 Aug 1;15(4):121. doi: 10.3390/jox15040121 (PMC12387716; doi:10.3390/jox15040121)
Supplement: Supplementary file 1 [file jox-15-00121-s001.zip › jox-3714386-supplementary.pdf]

PRISMA 2020 Checklist

Section and Topic	Item #	Checklist item	Location where item is reported
**TITLE**			
Title	1	A systematic review on contamination of marine species by chromium and zinc: effects on animal health and risk to consumer health	1
**ABSTRACT**			
Abstract	2	Potentially toxic elements, such as chromium (Cr) and zinc (Zn), play an essential role in human and animal organisms. However, some harmful effects of excessive exposure to these elements through food are still unknown. In this sense, this study aimed to evaluate the anthropogenic contamination of chromium and zinc for aquatic biota and seafood consumers. Based on the PRISMA protocol, 69 articles were carefully selected to compose this systematic review. The main results point to a wide distribution of these elements, which have familiar emission sources, in the aquatic environment, especially in highly industrialized regions. Significant concentrations of both have been reported in different fish species, which sometimes represent a non-carcinogenic risk to consumer health and a carcinogenic risk related to Cr. New studies should be encouraged to fill gaps such as the characterization of the toxicity of these essential elements through fish consumption; determination of limit concentrations updated by international regulatory institutions, especially for zinc; studies on the influence of abiotic factors on the toxicity and bioavailability of elements in the environment and those that evaluate the bioaccessibility of these elements in a simulated digestion system, when in high concentrations.	1
**INTRODUCTION**			
Rationale	3	Taking into consideration the side effects presented by the ingestion of high levels of these two essential elements to the human body and the increasing level of toxic waste containing them being released into the environment, it's necessary to analyze the levels present in the fish products being consumed, as marine animals are affected the most by the elevated levels of effluents. It is worth highlighting that the choice to evaluate these two metals in particular is related to common sources of emission and the lack of information regarding these elements of such importance to health, but with toxic potential.	2-3
Objectives	4	What are the primary sources of contamination of the metals studied (Zn and Cr)? Which chemical species of the elements is mainly responsible for the contamination? What risks are presented by Zn and Cr to the final consumer of the contaminated fish products? The presence of contaminants found was enough to alter the organisms observed?	3
**METHODS**			
Eligibility criteria	5	Abstracts and paper were removed if the papers did not investigate associations between animal/matrix (marine species) and the presence of Zinc (Zn) and Chromium (Cr). Thus, four criteria were selected for articles to be excluded from the selection: not studying marine species (Reason 1), or works on the freshwater environment (Reason 2), studies only on soil or sediment, without correlation with water or animals (Reason 3), works that addressed the results superficially, without clear justifications or results that stood out in relation to other works that managed to carry out a more in-depth approach (Reason 4). Some studies considered essential but not found in any research bases were added, such as those that address complementary data on chemical species of the elements studied and their mechanisms on the evaluated organisms.	3
Information sources	6	A literature search was performed using Medical Subject Headings (MeSH) terms in the PubMed, Web of Science, and Embase databases. The screening process was performed from March to May 2025.Further directed searching was also carried out by checking the reference list of relevant articles. Search Component 1 (SC1) - Population search: “Fish OR crab OR lobster OR muscle OR “swimming crab” OR squid OR “marine species” OR shrimp OR bivalve OR crustacea OR Seafood”. Search Component 2 (SC2) - Intervention search: “Zn OR zinc OR Cr OR chromium”. Search component 3 (SC3): “Biomagnification OR bioaccumulation OR “health risk” OR contamination OR “Risk assessment” OR “human health risk” OR “environmental contamination”. After retrieving the Search Component results, the Boolean operator “AND” was used to combine SC1, SC2, and SC3.	3
Search strategy	7	Following four sequential stages, the two authors (A.M.R.F and P.A.R) first independently conducted a preliminary selection of identified abstracts and paper titles. The search was limited to English and publishing dates between 2012 and 2025. Editorials, letters, reviews, mini-reviews, and Ph.D. theses were excluded. Further directed searching was also carried out by checking the reference list of relevant articles. Search Component 1 (SC1) - Population search: “Fish OR crab OR lobster OR muscle OR “swimming crab” OR squid OR “marine species” OR shrimp OR bivalve OR crustacea OR Seafood”. Search Component 2 (SC2) - Intervention search: “Zn OR zinc OR Cr OR chromium”. Search component 3 (SC3): “Biomagnification OR bioaccumulation OR “health risk” OR contamination OR “Risk assessment” OR “human health risk”	3

Section and Topic	Item #	Checklist item	Location where item is reported
		OR "environmental contamination". After retrieving the Search Component results, the Boolean operator "AND" was used to combine SC1, SC2, and SC3. After the search, the articles were grouped using the Zotero program and duplicates were excluded. The next step involved reading the titles and abstracts, excluding articles that did not meet the research objectives and met the exclusion criteria. Then, the remaining articles were read in full and those that did not meet the research objectives and met the exclusion criteria were eliminated, leaving the 69 articles that were used in this review.	
Selection process	8	The studies chosen for use in this article needed to contain information that answered one or more of the research questions (2.1. Focus Question), and therefore, they had to meet the research objectives. Therefore, they also had to not meet the established exclusion criteria. All articles were evaluated individually by the authors and both followed the same criteria to establish the documents that would be included and excluded.Data for each article were collected individually by the authors, seeking to extract the same standardized information from each document. This information was then extracted into a spreadsheet, which was subsequently checked by all authors to confirm the information. For all articles, data on the study site, species evaluated, metal concentration, risk assessment results or measured abiotic parameters, metal chemical species, and main observed changes were extracted.	3
Data collection process	9	Specify the methods used to collect data from reports, including how many reviewers collected data from each report, whether they worked independently, any processes for obtaining or confirming data from study investigators, and if applicable, details of automation tools used in the process. Data for each article were collected individually by the authors, seeking to extract the same standardized information from each document. This information was then extracted into a spreadsheet, which was subsequently checked by all authors to confirm the information. For all articles, data on the study site, species evaluated, metal concentration, risk assessment results or measured abiotic parameters, metal chemical species, and main observed changes were extracted.	3
Data items	10a	The data evaluated in all articles were: study site, species evaluated, metal and its chemical species, element concentration, result of the non-carcinogenic risk assessment and carcinogenic risk (if present), water parameters measured (if evaluated), changes caused in animal and/or human health.	3
	10b	Characteristics that were considered fundamental to elucidate the results collected from each article and that were not in the primary list were added as observations. Articles that did not present consistent information, results that could be compared and without justifications were excluded.	3
Study risk of bias assessment	11	Possible sources of bias include inclusion/exclusion criteria, the impact of missing data, and missing primary results. A preliminary search was conducted by all authors in multiple databases and languages to minimize selection bias, covering various article types and publication years. This exploratory phase allowed for identifying knowledge gaps and helped select inclusion and exclusion criteria that aligned with this review's objectives. Furthermore, all selected studies were assessed for the quality of the data presented, the clarity in the discussion of the results, and the presentation of a well-described methodology appropriate for the study's purpose. All studies were carefully evaluated, and those that did not present consistent results, discussions and methods, when necessary, were excluded or contextualized in the debate to avoid distortions in the risk estimates.	4
Effect measures	12	Study site (location-city and country), species evaluated (fish, crab, mussel, squid, shrimp, gastropod), metal and its chemical species (Cr, Zn and their inorganic species), element concentration (in mg kg^{-1}), result of the non-carcinogenic risk (EMI/EDI and THQ) assessment and carcinogenic (CR) risk (if present), water parameters (pH, salinity, temperature, oxygen) measured (if evaluated), changes caused in animal and/or human health (morphological changes, biochemical changes, symptoms).	
Synthesis methods	13a	Each study was assessed for the presence of elements that indicated the paper met the aforementioned eligibility criteria. Each element was collected and tabulated, and the data set was assessed together.	3
	13b	Regarding data preparation, the concentration of the tabulated elements was converted to a single unit of measurement and wet weight (mg.kg^{-1} w.w.).	3
	13c	Describe any methods used to tabulate or visually display results of individual studies and syntheses. The results were tabulated using Excel and presented visually in Table 1, with element concentrations and figures relating to geographic distribution (Figure 3), trophic niche (Figure 4), and species vulnerability status (Figure 5).	5-11

Section and Topic	Item #	Checklist item	Location where item is reported
	13d	The articles selected for this review were grouped and evaluated using Zotero® software, and data extracted from the articles were tabulated using Excel. Graphs were created using R® 4.3.1.	
**RESULTS**			
Study selection	16a	A total of 1,629 articles were identified in PubMed, 1,594 in Embase, and 84 in Web of Science, totaling 3,307 articles. Of these, 595 were duplicates. A total of 2,712 articles remained after the exclusion of repeated articles. After reading the titles and abstracts, 2,362 articles were excluded because they were not aligned with the study's objective, leaving 350 articles. After reading the manuscripts in full, 46 studies that met the study's aim and met the selection criteria were selected. In the next stage, 23 articles were added due to the complementarity of data on the chemical species of the elements studied, their mechanisms in the evaluated organisms (15), and regulatory information regarding the permitted limit of each element (7). Therefore, the total number of studies used to compose the article was 69 (Figure 1). Among the reasons for excluding articles are those that did not study marine species (Reason 1= 458) or works on the freshwater environment (Reason 2= 107), those that dealt only with soil or sediment, without correlation with water or animals (Reason 3= 277), works that superficially addressed the results, without clear justifications or results that stood out compared to other works that managed to carry out a more in-depth approach (Reason 4= 1,824).	4
	16b	In general, studies that answered the research questions in some way but did not provide in-depth discussion and provide justification for the results were excluded.	4
Study characteristics	17	The studies included in the review were those that quantified Cr and Zn concentrations in marine animal samples (table 1). Preferably, those that also quantified them in water samples, assessed correlations with abiotic factors, and performed a health risk assessment were also included (4.6. Consumer Health Risk Assessment). Studies that conducted toxicological tests to investigate health effects were also selected (*4.2. Maximum Cr and Zn levels and the response in aquatic animals*). Other characteristics extracted from the studies were their geographic locations, trophic niches and the vulnerability of the species (Figure 3, 4 and 5 respectively).The risk of bias is presented in supplementary material 1	5-14
Risk of bias in studies	18	The risk of bias is presented in supplementary material 1	20
Results of individual studies	19	Table 1. Concentration (mean or range - minimum and maximum) or mean concentration of Zn and Cr (mg/kg w.w.) found in different marine species	7-11
Results of syntheses	20a	In general, the articles that studied only these elements were related to toxicity studies or their dynamics in the environment. Only a few studies have focused on one organism; most have studied various species. The animals analyzed during the literature search were fish, mussels, crabs, shrimp, shark, gastropod and squid. The observed studies were identified in several parts of the world (Figure 3), especially in estuarine regions with a rich biodiversity and breeding grounds for many marine species. The studies were mainly concentrated on the Asian continent, emphasizing populous and highly industrialized countries (China and India), followed by the European continent, with studies in areas of lower anthropogenic impact and environmental protection (the Canary Islands). The African continent was also studied, with work in the northern (Algeria) and southern region (South Africa), and only one study was conducted on the American continent, specifically in the Galapagos Islands, Ecuador, which is also an environmental protection area. The articles selected to compose this review mainly sought to quantify several elements, including Cr and Zn in commercially important fish species, molluscs and crustaceans. It was possible to classify the fish species according to the International Union for Conservation of Nature (IUCN) Red List of Threatened Species. In this classification, species can be divided into not evaluated, insufficient data, least concern, near threatened, vulnerable, endangered, critically endangered, extinct in the wild and extinct. No data were found regarding the other animal groups covered in this study, only regarding fish (Figure 5).	4-11
Reporting biases	21	The risk of bias is presented in supplementary material 1	20
Certainty of evidence	22	N/A	
**DISCUSSION**			
Discussion	23a	The concentrations found in the different studies were generally below the limits and did not represent a risk to consumer health. However, some	21

Section and Topic	Item #	Checklist item	Location where item is reported
		studies demonstrated the presence of significant concentrations and indicated both a non-carcinogenic risk related to zinc and a carcinogenic risk associated with Cr.	
	23b	Discuss any limitations of the evidence included in the review.New studies should be encouraged to fill gaps such as the characterization of the toxicity of these essential elements through seafood consumption; determination of limit concentrations updated by international regulatory institutions, especially for zinc; studies on the influence of abiotic factors on the toxicity and bioavailability of elements in the environment and those that evaluate the bioaccessibility of these elements in a simulated digestion system, when in high concentrations.	21
	23c	Some limitations are related to the selected databases and the inclusion and exclusion criteria.	21
	23d	The data presented in this article demonstrate how essential it is to continuously monitor contaminant levels in large bodies of water and their biosphere, since many studies have shown increasing levels of chromium and zinc not only in places affected by human action, but now also in environments considered protected, such as the Canary Islands and several estuaries in Europe. This fact is highly worrying, since these breeding grounds for many marine species that, as demonstrated in this article, have their reproduction affected by metallic contaminants.	21
**OTHER INFORMATION**			
Registration and protocol	24a	The article has been registered with the OSF Registries.	21
	24b	The protocol can be accessed via the website https://www.cos.io/products/osf-registries	21
Support	25	The authors are thankful for the financial support provided by the Fundação de Amparo à Pesquisa do Estado do Rio de Janeiro (FAPERJ) Brazil — grant number [E- 26/200.891/2021 (C.A.C.J.)], the Conselho Nacional de Desenvolvimento Científico e Tecnológico (CNPq) - grant number [313119/2020-1 (C.A.C.J.); 175429/2023-5 (PAR)], and the Coordenação de Aperfeiçoamento de Pessoal de Nível Superior (CAPES) Brazil —Finance Code 001 and for granted scholarship to A.M.R.F.	21
Competing interests	26	The authors declare no conflicts of interest.	21
Availability of data, code and other materials	27	The data analyzed in this article were extracted from other works and compiled, and can be verified upon request to the authors.	21

From: Page MJ, McKenzie JE, Bossuyt PM, Boutron I, Hoffmann TC, Mulrow CD, et al. The PRISMA 2020 statement: an updated guideline for reporting systematic reviews. BMJ 2021;372:n71. doi: 10.1136/bmj.n71. This work is licensed under CC BY 4.0. To view a copy of this license, visit https://creativecommons.org/licenses/by/4.0/
